# Best Practices in Academic Mentoring: A Model for Excellence

**DOI:** 10.1155/2012/937906

**Published:** 2012-05-23

**Authors:** Jan M. Nick, Theresa M. Delahoyde, Darlene Del Prato, Claudia Mitchell, Jennifer Ortiz, Clarise Ottley, Patricia Young, Sharon B. Cannon, Kathie Lasater, Deanna Reising, Linda Siktberg

**Affiliations:** ^1^School of Nursing, Loma Linda University, Loma Linda, CA 92350, USA; ^2^Department of Nursing, Saniku Gakuin College, Otaki-machi, Chiba-ken 298-0297, Japan; ^3^School of Nursing, BryanLGH College of Health Sciences, 5035 Everett Street, Lincoln, NE 68506, USA; ^4^Department of Nursing and Health Professions, State University of New York Institute of Technology, 100 Seymour Road, Utica, NY 13502, USA; ^5^College of Nursing, University of Cincinnati, 3110 Vine Street, Cincinnati, OH 45221-0038, USA; ^6^School of Nursing, Suffolk County Community College, 533 College Road, Selden, NY 11874, USA; ^7^Department of Nursing, Shepherd University, P.O. Box 5000, 301 North King Street, Shepherdstown, WV 25443-5000, USA; ^8^School of Nursing, Minnesota State University Mankato, 360 Wissink Hall, Mankato, MN 56001, USA; ^9^School of Nursing, Texas Tech University Health Sciences Center, 800 West4th Street, Odessa, TX79763, USA; ^10^School of Nursing, Oregon Health & Science University, 3455 SW Veterans' Hospital Road, Portland, OR 97239, USA; ^11^School of Nursing, Indiana University, 1033 East Third Street, Bloomington, IN 47405, USA; ^12^School of Nursing, Ball State University, Muncie, IN 47306, USA

## Abstract

Mentoring is important for the recruitment and retention of qualified nurse faculty, their ongoing career development, and leadership development. However, what are current best practices of mentoring? The purpose of this paper is to provide an overview of a model for excellence in establishing a formal mentoring program for academic nurse educators. Six themes for establishing a formal mentoring program are presented, highlighting best practices in mentoring as culled from experience and the literature. Themes reflect aims to achieve appropriately matched dyads, establish clear mentorship purpose and goals, solidify the dyad relationship, advocate for and guide the protégé, integrate the protégé into the academic culture, and mobilize institutional resources for mentoring support. Attending to the six themes will help mentors achieve important protégé outcomes, such as orientation to the educator role, integration into the academic community, development of teaching, scholarship, and service skills, as well as leadership development. The model is intended to be generalizable for faculty teaching in a variety of academic nursing institution types and sizes. Mentoring that integrates the six themes assists faculty members to better navigate the academic environment and more easily transition to new roles and responsibilities.

## 1. Introduction

Mentoring is important for the recruitment and retention of qualified nurse faculty, their ongoing career development, and leadership development. The functional outcomes of mentoring encompass orientation to the educator role, integration into the academic community, development of teaching, scholarship, and service skills, as well as leadership development [1]. Given the increasing shortage of experienced nurse educators, faculty may be challenged in finding a mentor and also in sustaining a mentoring relationship. In addition to the above issues, in general, questions exist around mentoring, such as the following. What are current best practices of mentoring? How can academic institutions support the mentoring process in order to develop and retain novice faculty during this time of economic hardship? How can nursing programs mitigate the challenges of academic mentoring?

The above questions are examples of some of the inquiries that motivated the authors to conduct an integrated review to design a template for excellence in mentoring in the context of nursing education. The authors aimed to create a template addressing the “what” and “how” of mentoring that would serve as a standardized best-practice model targeting faculty across the career span. This paper reflects the yearlong work of the group project from the fourth cohort and Project Director of the National League for Nursing (NLN)/Johnson & Johnson (J&J) Faculty Leadership and Mentoring Program. The NLN, with generous support from J&J, established a faculty leadership and mentoring program in 2007 with the overall goal to prepare leaders to transform the future of nursing education [2]. Each year, program participants consisted of five experienced nurse leaders (mentors), five emerging nursing leaders (protégés), and the program leader. The mentors and protégés were matched based on experience and interest. In consultation with their mentors, protégés chose individual leadership projects to work on throughout the year; additionally, the 10 participants also worked on one group project. The fourth cohort from the program focused on how formal mentoring could transform nursing education and expanded the initial work by the NLN [1].

 The word “mentor” derives from Greek mythology when Odysseus entrusted the care of his son to his friend “Mentor,” to serve as guide and teacher while he went to fight the Trojan War [3]. Since then, the concept of mentoring has evolved into a multidimensional interactive process that can be formal or informal and evolves over time according to the needs and desires of the mentor and protégé [4]. Haggard et al. define mentoring as a one-to-one reciprocal relationship between a more experienced and knowledgeable faculty member (the mentor) and a less experienced one (the protégé). The relationship is characterized by regular/consistent interaction over a period of time to facilitate protégé development [5].

Research indicates many positive outcomes as a result of mentorship. For example, when a novice educator is formally mentored by a more experienced and accomplished academician, the novice educator more quickly assumes the full scope of the academic role and is more productive [6]. Across settings, mentoring has contributed to higher career satisfaction and increased departmental or organizational morale [7, 8]. Mentored faculty reported augmented professional identity and experienced a smoother bridge from practice to the academic environment [7]. In addition, mentored faculty reported increased self-confidence and professional development [9]. Not surprisingly, institutions have benefitted from sponsoring faculty mentoring programs by experiencing improved retention rates [7, 10, 11] and increased productivity in the workplace [7, 9].

Often, nurse educators enter the academic role without a clear idea of the full scope of their responsibilities, or how they can actually achieve them at a level sufficient to become productive academicians. Others labor under the misconception that teaching is the academicians' primary responsibility. Mentoring relationships can help educators understand the multifaceted roles of an academician, which facilitates achieving success in a timely manner in the areas of teaching, scholarship. and service. Research demonstrates that careers did not progress as satisfactorily when faculty did not have mentors, compared to those who did [6, 9, 12]. Unfortunately, many novice academicians cannot avail themselves of mentoring opportunities, because formal mentoring programs are not common in the nursing education organizational culture.

The purpose of this paper is to provide an overview of a model for excellence in establishing a formal mentoring program for academic nurse educators. The model is intended to be generalizable for faculty teaching in a variety of academic nursing institution types and sizes.

## 2. Methods

The authors participated in a formal distance mentoring program and determined to engage in a heuristic inquiry to study mentoring. Initially, the authors used an inductive process to identify mentoring themes. At a face-to-face meeting, each participant shared lived experiences (good or bad) of either being mentored or mentoring someone and described the significance of the experience. The group discussed practices of mentoring revealed in each story; a recording secretary listed practices that group members agreed upon. Through reflection and dialogue, the group clustered 25 original practices into six categories based on similar thematic content. The six categories served as the basis for six in-depth reviews of the literature; refinement of the categories occurred over several months during exploration of research literature. Databases searched included Academic Search Elite, CINAHL, ERIC, PUBMED, Google, and Google Scholar. Search terms included mentor, protégé, mentee, mentoring, faculty mentoring programs, mentorship, mentorship advocacy, collegiality, academic networking, academic socialization, matching, resources, and workload release. Only articles in English were considered for the integrated review.

## 3. Findings and Discussion

**Figure 1:**
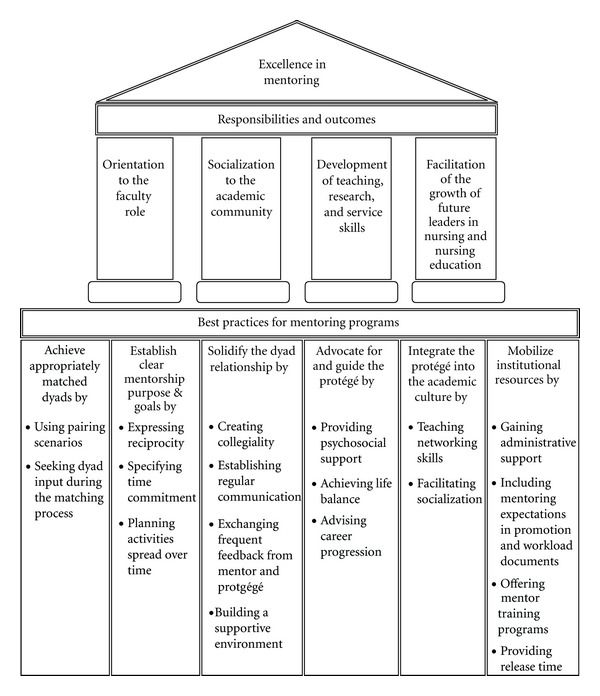
The model: *Best Practices in Academic Mentoring: A Model for Excellence.* Fourth Cohort, NLN/Johnson & Johnson Faculty Leadership and Mentoring Program.

Six categories reflecting aims for establishing a formal mentoring program are presented, highlighting six best practices in mentoring. These practices support the four pillars of excellence (Figure 1). The discussion further describes how the best practices collectively form a model of mentoring excellence.

### 3.1. Achieve Appropriately Matched Dyads

Appropriate fit is an important aspect for creating a successful mentor/mentee (protégé) relationship; therefore, matching the right mentor to the right protégé is the first best practice theme. Pairing can be accomplished by a variety of methods described below. There is, however, no clear evidence as to which method is best. Fortunately, the literature does give guidance that seeking individual input from both the mentor and protégé will result in the best fit.

#### 3.1.1. Using Pairing Scenarios

The mentor and protégé are often referred to as a dyad or pair. There are five basic ways that mentors and protégés can come together to form a mentoring dyad: (a) paired administratively based on arbitrary criteria, (b) paired administratively based on specified criteria, (c) paired based on protégé selection of mentor, (d) paired based on mentor selection of a protégé based on recognized potential and a desire to “take under wing,” and (e) paired based on finding each other and creating their own dyad relationship. Formal mentoring programs tend to use one of the first three approaches to pair mentor and protégé [13] which means that often, the protégé and mentor inputs are not taken into account in the pairing process [14].

Clearly, administrators often assign mentors based on availability: whose turn it is, who is most friendly, or who is coteaching a course. While this approach is common practice in contemporary nursing education, it may not produce the best fit. Mentor-protégé mismatch has been identified as a common problem in formal mentoring programs from both perspectives [15]. Differences in background, age, personality, and interests can lead to mismatched perceptions [16]. However, there is no clear evidence about the effectiveness of using one matching scenario over another. There is, however, evidence that having protégés and/or mentors provide input in the matching process results in better match outcomes. Therefore, obtaining input to achieve appropriate matching cannot be overemphasized.

#### 3.1.2. Seeking Dyad Input during the Matching Process

The significance of obtaining dyad input during the matching process has been noted by many researchers [17]. Allen et al. [18] found that when mentors provide input during the matching process, mentors demonstrated stronger commitment to the relationship, and had a greater understanding of the mentoring program. They also perceived the relationship to be of better quality and provided more career advice to the protégé. Another study by Allen et al. further reported that protégé input into the match was associated with greater mentorship quality, career mentoring, and role modeling while mentor input into the match was associated with greater mentorship quality and career mentoring [19]. Eby and Lockwood [16] showed that mentors desired more information about how matches were made—as mentors perceived the assignment as being haphazard when they were not clear how the matching process occurred.

Parise and Forret [20] studied the relationship between voluntary participation, mentor input to the matching process and training, and the benefits (or costs) perceived by mentors. Voluntary participation was positively related to rewarding experiences. Conversely, mentor input into the matching process was negatively related to what the authors called nepotism, that is: the less input given in the matching process, the more the mentor perceived favoritism occurred during the assignment. On the other hand, group interview data showed most mentors believed it was more important for protégés to have input into the selection process than mentors since it was a job expectation, and they should accept whomever they were given. Additionally, mentors felt it was up to the protégé to determine what was needed. The mentors' only concern was in their own ability to provide a good mentoring experience for a protégé. This last comment supports the concept of mentor training, discussed in a later section.

One strategy to elicit input into a matching process is to match the dyad using criteria—in this way, the potential for compatibility increases. Headlam-Wells et al. [21] tested criterion-based pairing in their research. The authors designed and used 11 criteria (age, number of years of work experience, level of qualification, marital status, children, dependent care, life/career history, personal skills, professional skills, vocational sector, and personal values) to pair up mentors and protégés. Matches were based on a majority of same responses by both mentor and protégé. Most importantly, protégés ranked their top three criteria in order of importance. The top two were having a mentor who could help with professional and personal skill development, and the third was being matched with a mentor with similar values. At the end of the mentorship, satisfaction ratings regarding the matchup showed 75% of mentors and 80% of protégés felt they were either “very well” or “quite well” matched. An example of this method, albeit on a simpler scale, is how the mentors and protégés were matched in the annual National League for Nursing/Johnson & Johnson Faculty Leadership and Mentoring program [2]. The application process required protégés to identify leadership interests, needs, and goals and ascertained mentors' experience and expertise, and then applicants were matched accordingly. Twenty dyads were successfully matched over four years using this basic approach.

Another strategy that encourages input of both mentor and protégé is a variation on speed dating. Berk [13] recommended using this concept with a novel application—speed mentoring—where several mentors and protégés meet briefly to form first impressions and then request to be matched to a specific person. When mentor requests match protégé requests, a dyad can be formed. This approach may be more useful with a large number of people.

Bozeman and Feeney [14] offered a third approach to facilitate successful matching of mentor and protégé. Their “goodness of fit” model identified three categories of factors to optimize social exchange within a dyad. When matching mentor-protégé pairs, using these factors will create the best fit. Categories include:

- endowments (e.g., knowledge, experience, and communication abilities);
- mentoring content (e.g., professional contacts and historical insider knowledge of office politics);
- preferences (e.g., value of modes of communicating, teaching; or learning).

The notion of goodness-to-fit generates questions for future research on how preferences affect endowments and endowments affect preferences. The fit will shape mentorship outcomes. For example, the dyad fit is good when “the mentor has the knowledge preferred by the protégé, and the ability to transmit that knowledge effectively…and the protégé has the ability and skill to fully [grasp] the knowledge being transmitted” [14, page 473].

Regardless of the strategy employed, the recommended best practice to achieve appropriately matched dyads is to obtain input in the matching process. Once dyads have been matched using input by both parties, the next best practice is to establish clear purposes and goals for the mentoring relationship. The following section provides direction as to what mentors should aim for when starting out in a new mentoring relationship.

### 3.2. Establish Clear Mentorship Purpose and Goals

Once paired, the dyad must clearly articulate the purpose of the mentorship relationship and set initial goals early on to give direction and clarity of future responsibilities. In addition to helping the protégé establish personal career goals, Chao [17] recommended mentors to take into consideration organizationally prescribed goals. When needs and desires guide the mentoring relationship, successful outcomes are more likely. The purpose and goals may be as broad as leadership development [2] or as focused as writing a research grant. Three expectations must be expressed early on in the relationship: (a) reciprocity, (b) time commitment, and (c) planning growth activities.

#### 3.2.1. Expressing Reciprocity

The concept of reciprocity occurred frequently in the mentoring literature. Sorcinelli and Yun [22] called this concept “creating a reciprocal partnership.” Wilson et al. [23] termed it “reciprocal learning”; while Carey and Weissman [3] labeled it “reciprocal relationship.” All three convey the idea that each party has the experience of being the giver and the receiver. Significant to the idea of reciprocity is that mentors must also identify their purpose for being mentors and articulate personal goals; that is, what they hope to derive from the relationship. Ideally, mentors must also perceive receiving benefits from the relationship. Benefits could be as intangible as exchange of ideas and input to more tangible as collegial work benefitting both participants. To have reciprocity identified clearly at the outset will foster the commitment to creating the relationship.

#### 3.2.2. Specifying Time Commitment

 Formal mentoring programs are characterized by a clearly stated timeframe for the dyad relationship that focuses on the protégé's development [21]. A typical duration for a dyad relationship in a formal mentoring program is one year [2, 21]. Clarifying the anticipated duration of the relationship and time commitment when the relationship is initiated is imperative so that goals can be achieved in a realistic manner [24]. In this manner, unmet expectations and disappointments are diminished.

#### 3.2.3. Planning Activities Spread Over Time

 Ideally, activities should be spread over time. While dyads have freedom to negotiate how they will communicate with one another, the key point is that both the mentor and the protégé are committed to and engaged in the mentoring relationship and participate in multiple activities over time [2, 25]. Regular interaction spread over time serves to connect and solidify the dyad relationship, another aim in the establishment of a formal mentoring program.

### 3.3. Solidify the Dyad Relationship

The formation of a relationship between mentor and protégé is crucial to effective mentoring. As the relationship unfolds and expectations are clarified, the dyad must strive to deepen the relationship. The relationship may be further developed through four strategies, namely, (a) creating collegiality, (b) establishing regular communication, (c) exchanging regular feedback from mentor and protégé, and (d) building a supportive environment.

#### 3.3.1. Creating Collegiality

Once the dyad has been matched appropriately and goals set, the next step in creating successful mentoring is for the pair to establish a collegial relationship. Collegial and reciprocal relationships are the basis for effective mentoring which ultimately fosters the protégé's academic success [23]. Collegiality in academia is seen most broadly when faculty demonstrate “cooperation and collaboration in a spirit of teamwork” [26]. Fischer [27] expounds on this theme by calling for institutions to establish “codes of conduct” so this cooperation and collaborative teamwork can be everyone's responsibility to implement (paragraphs 29, 30). A prime strategy for cultivating a meaningful collegial mentor-protégé relationship is establishing mutual respect and trust; without these two, neither a collegial nor a collaborative relationship can exist [28]. This aspect alone has strong implications for departments desiring to establish mentoring programs. Without solid collegial relationships, mentoring programs will struggle. Interventions aimed at building trust and respect may need to take precedence prior to implementing a mentoring program.

Organizations have used multiple strategies for recruitment and retention of faculty. Whereas, financial incentives and promotional campaigns are effective in recruiting faculty in the short term, they do not necessarily retain faculty in the long term. Emerging evidence suggests that an environment of incivility in the workplace is often the reason for employee attrition [29, 30]. In fact, nurse educators who experience incivility are more likely to leave their employment and sometimes leave nursing altogether [31, 32]. Improving the workplace environment primarily through establishing collegial relationships has been one of the most effective strategies in faculty retention [33]. Through the development of guidelines for establishing healthy civil and supportive work environments, professional nursing education organizations are promoting mentoring as a means of faculty retention [34, 35].

#### 3.3.2. Establishing Regular Communication

 To solidify a relationship, regular “connectedness” cannot be understated. The more formalized and set the pattern of communication is the better the connection occurs between the pair. Smith and Zsohar [11] and White et al. stressed the importance of establishing negotiated times for regular communication that fits the personalities of both parties [24]. Planned activities, such as regular journaling, workshops, and off-campus activities, facilitated the development of meaningful relationships [2, 24]. What appears significant is that the method of communication is not as important as the regularity.

#### 3.3.3. Exchanging Frequent Feedback from Mentor and Protégé

 Mentors can be successful resources when dyads prepare for relationships by not only reflecting on and defining goals, but also identifying challenges and asking for feedback to evaluate relationship effectiveness [23, 36–39]. Allen et al. demonstrated that protégés who asked for and were accepting of feedback received higher quality and quantity of feedback from their mentors [19]. Receiving feedback is sound since these same authors reported that frequency of feedback from mentors was strongly associated with increased protégé productivity.

#### 3.3.4. Building a Supportive Environment

 Creating an environment where the protégé feels supported cannot be overemphasized; this action has a direct effect on solidifying the dyad relationship [11]. When protégés experience support, they feel free to exercise independent thinking, a willingness to be creative, to offer ideas for consideration, and verify lines of reasoning with their mentors. Unsupportive environments hinder the protégé's willingness to be open, take risks, and collaborate. Smith and Zsohar [11] found the ability to create collegiality was directly impacted by the presence or absence of a supportive environment.

What does a supportive environment look like? Mentors who show positive regard and genuine caring are willing to listen, display empathy and trustworthiness, give encouragement, provide authentic feedback, and create supportive environments [6, 25, 40]. Once the dyad relationship solidifies, the mentor is ready to display confidence in his or her ability to advocate for and guide the protégé. In turn, the protégé is ready to trust the mentor's judgment and recommended actions. Advocating for the protégé is another aim in the establishment of an excellent formal mentoring program.

### 3.4. Advocate for and Guide the Protégé

The fourth theme of best practices is advocating for and guiding the protégé. Although much of the research in this area is less recent, it is relevant to review. Advocacy provides tangible benefits for the protégé throughout the mentoring process. An advocate is someone who supports another and acts for another's benefit or one who speaks for another's behalf. The literature provides many areas for mentor advocacy/guidance. This paper, however, focuses on three primary strategies: (a) providing psychosocial support, (b) achieving life balance, and (c) advising career progression.

#### 3.4.1. Providing Psychosocial Support

 With multifaceted academic responsibilities, faculty may easily feel pulled in many directions and become discouraged. Earlier research indicated that a major aspect of advocacy is for mentors to attend to psychosocial undertakings [9, 41, 42]. Aagaard and Hauer [41] studied activities of physician mentors; 98% of mentors identified *motivating protégés* as one of the top activities, followed by 91% providing *moral support*. Protégés who were queried about mentor activities ranked being acknowledged as a person and as a professional very highly (4.8 of 5), while having mentors who listened to their expressed concerns ranked a perfect 5 of 5 [42]. Clearly, this aspect of advocacy is highly valued by both mentors and protégés.

#### 3.4.2. Achieving Life Balance

 Achievement of life balance between personal and professional work is critical to the success of a new faculty member. Straus et al. [43] saw the mentor as advocating for the protégé and provided guided decision making so that protégés can learn time management strategies. The mentor can advise the protégé on how to work smarter, not harder; guiding the protégé to create boundaries so that professional activities do not blur into personal time is essential to achieve equilibrium. Boice [44] reiterated the importance of life balance over and over again. Providing strategies for new faculty members to be productive, yet still maintaining a reasonable work week, is of prime importance. An interesting result is that the mentor can also work to achieve life balance, an issue many seasoned faculty struggle with as well.

#### 3.4.3. Advising Career Progression

 An obvious area for protégé advocacy/guidance is advising career progression while helping the protégé achieve balance between personal and professional responsibilities. Mentors often promote progress by helping protégés set professional goals, mapping a career plan, and establishing clear career milestones [9, 43]. Mentors can also help identify career advancement opportunities the protégé may not be aware of or know how to find [41]. The literature clearly articulated the impact of no mentoring; for example, nonmentored faculty often struggle with scholarship over their entire career [9, 12, 41, 42, 44, 45]. The implication of these findings is that faculty must be cognizant of institutional alignment with scholarly productivity. If a faculty member feels strongly about establishing a productive research program, finding an institution with a mentoring program is one indicator of the institution's commitment to scholarly productivity. Conversely, trying to accomplish scholarly productivity in an institution that fails to offer a mentoring program may indicate the structure for productivity is not sufficiently established.

### 3.5. Integrate the Protégé into the Academic Culture

The fifth theme of best practices offers two separate but critical activities that support the program aim of integrating protégés into the academic culture: (a) teaching networking skills and (b) facilitating socialization to the academic culture. The value of integrating protégés into the academic culture is that it allows mentors to share intellectual capital and emotional intelligence. The mentor should give due diligence to this theme, since these qualities affect the protégé's ability to become a productive member of academe.

#### 3.5.1. Teaching Networking Skills

 Mentors can facilitate protégé integration into the culture of academe by helping protégés learn to network and establish professional contacts. Coleman et al. identified networking as extremely important for getting to know professional contacts in the clinical area [12]. Networking offers the potential for connecting with people in a particular field that, later in time, may assist with career advancing opportunities. Boice [44], whose research spans 30 years, stated that the “strongest predictor for early success in an academic career was that new faculty found social supports and networks” (page 220). The protégé may consider identifying internal and external mentors since the benefit obtained from each may differ. For example, the internal mentor can assist the protégé with integration into the institutional culture, while a mentor external to the institution can help the protégé identify networking opportunities on local, national, or international levels [11, 46]. Often, the mentor can invite the protégé to collaborate on projects and introduce the protégé to an expanded networking circle. These activities have been shown to promote professional development and increase scholarly productivity [23, 24].

#### 3.5.2. Facilitating Socialization

A second way mentors can facilitate protégé integration into the culture of academe is by helping protégés navigate the social structure and culture of academics. Faculty new to academia are rarely prepared educationally or experientially for the multiple roles and expectations as well as the isolation that may present itself with academe—the reason being, academia is an unfamiliar culture to them [1]. There is a strong correlation between organizational support during the career-entry stage and the stress novice employee's experience every day.

A smooth transition into the academic culture by a novice educator may not be realized without the assistance of a mentor since the social norms and expectations are most often not written—or easily understood [47]. Socialization, therefore, becomes crucial for new faculty to feel they are members of the academy. Importantly, Smith and Zsohar [11] showed that mentors who facilitate protégé socialization have a direct impact on the quality of nursing education provided. It is the mentor who helps socialize and prepare novice faculty in the areas of teaching, research, and service.

### 3.6. Mobilize Institutional Resources

The sixth theme of best practices, mobilizing institutional resources, requires institutional responsibility in order for the intended mentoring program to thrive. Four strategies are identified here that reflect administrative, collegial, or financial investments.

#### 3.6.1. Gaining Administrative Support

 When people think of the term *resources*, they often think of money. Although money is a key component, the primary resource requirement for an effective formal mentoring program is administrative support and commitment. Therefore, a critical strategy to assist mobilizing resources in mentoring is to gain administrative support at both the departmental and college level prior to initiating any formal mentoring program [10, 48, 49]. Without authentic support from departmental administration, mentoring programs are likely to struggle.

#### 3.6.2. Including Mentoring Expectations in Promotion and Workload Documents

Another action that can be readily accomplished is the inclusion of mentoring activities into faculty expectations; these can be demonstrated in the criteria for promotion and tenure, salary merit, and workload calculation documents. Including expectations that experienced faculty mentor junior faculty in these documents sends a strong message of acceptance by both administration and faculty. Plus, faculty receive acknowledgement and credit for time spent mentoring other faculty.

The time is ripe for this action; a practice analysis conducted in 2005 by the National League for Nursing showed that, nationally, faculty and administrators identified mentoring as part of the role of nurse faculty [50]. It must be noted that protégés do not only have to be junior faculty. Cariaga et al. [51], as well as the National League for Nursing [1], showed that establishing a mentoring climate can increase productivity in faculty at the early-, mid-, and late-stage of the academic career ladder.

#### 3.6.3. Offering Mentor Training Programs

 Because effective mentorship skills do not always occur naturally, a third strategy is to train mentors. Effective mentorship extends beyond simply sharing one's knowledge or expertise; mentors can also be taught how to be effective mentors [39]. This best practice includes having a mentor-training workshop at institutions of higher learning with the purpose of increasing the number and quality of mentors. The expense for such a program can be distributed across departments since mentoring is important for all faculty.

#### 3.6.4. Providing Release Time

 This best practice requires some financial expense in the form of release time for mentor and protégé participants [49]. Release time however, pays for intangible activity at first. What administrators must remember is that although intangible at the beginning, release time has been shown to be directly associated with increased scholarly productivity and increased innovation by faculty [51]. Administrators and faculty should not be deterred over this last resource, since mobilizing financial resources can be seen as an institutional long-term return on investment.

## 4. A Model for Excellence in Mentoring

The model (see Figure 1) builds on the preliminary work conducted by the National League for Nursing in 2006 and includes four mentoring outcome pillars: orientation to the faculty role, socialization to the academic community, development of teaching, research, and service skills, and facilitation of the growth of future leaders in nursing and nursing education [1]. Our work introduces six themes of current evidence of best practice which underpin the four mentoring outcome pillars. The six major themes of best practices reflect the aims of establishing a formal mentoring program and include (a) achieve appropriately matched dyads, (b) establish clear mentorship purpose and goals, (c) solidify the dyad relationship, (d) advocate for and guide the protégé, (e) integrate the protégé into the academic culture, and (f) mobilize institutional resources. Attending to these six themes will help mentors achieve the four mentoring outcome pillars. The model can be used to create structure or serve as outcome measures for any mentoring program.

“Best practice” is operationally defined as those actions that produce the most desirable faculty outcomes, based on evidence and real life experiences. An underlying assumption is that relationships play a key role in any successful mentorship experience, as evidenced by the focus of the best-practice themes as it pertains to ways to initiate, build, solidify, advocate, or integrate the relationship.

## 5. Conclusions

Mentoring programs have many benefits and contribute to improved faculty morale, higher career satisfaction, and increased self-confidence in professional development. Mentored faculty publish more, obtain more grants, and are promoted more quickly. Institutions providing mentoring programs experience increased retention and improved sense of community and professional identity.

The model, “*Best Practices in Academic Mentoring: A Model for Excellence,*” provides a schema that can be used to create programs of mentoring and functions as a thematic basis for evaluation of program effectiveness. Mentoring assists faculty members to better navigate the academic environment and more easily transition to new roles and responsibilities. A work environment where collaborative and reciprocal peer and co-mentoring are present results in a rich, satisfying, and rewarding career experience for both mentor and protégé. It ultimately moves the profession forward.

Imagine the impact on faculty career attainment, institutional culture, the science of nursing, and leadership development in nursing education, if all faculty were mentored. Mentoring programs are especially important at a time when academia is experiencing a shortage of nurse faculty members. A trait of a true leader then is being an excellent mentor and developing future leaders.
